# Fully moderated t-statistic in linear modeling of mixed effects for differential expression analysis

**DOI:** 10.1186/s12859-019-3248-9

**Published:** 2019-12-20

**Authors:** Lianbo Yu, Jianying Zhang, Guy Brock, Soledad Fernandez

**Affiliations:** Center for Biostatistics, Department of Biomedical Informatics, The Ohio State University, 1800 Cannon Dr., Columbus, 43210 OH USA

**Keywords:** Fully moderated T-statistic, Linear mixed-effects model, Variance shrinkage, Expected number of false positives

## Abstract

**Background:**

Gene expression profiling experiments with few replicates lead to great variability in the estimates of gene variances. Toward this end, several moderated t-test methods have been developed to reduce this variability and to increase power for testing differential expression. Most of these moderated methods are based on linear models with fixed effects where residual variances are smoothed under a hierarchical Bayes framework. However, they are inadequate for designs with complex correlation structures, therefore application of moderated methods to linear models with mixed effects are needed for differential expression analysis.

**Results:**

We demonstrated the implementation of the fully moderated t-statistic method for linear models with mixed effects, where both residual variances and variance estimates of random effects are smoothed under a hierarchical Bayes framework. We compared the proposed method with two current moderated methods and show that the proposed method can control the expected number of false positives at the nominal level, while the two current moderated methods fail.

**Conclusions:**

We proposed an approach for testing differential expression under complex correlation structures while providing variance shrinkage. The proposed method is able to improve power by moderation and controls the expected number of false positives properly at the nominal level.

## Background

To understand the dynamics of gene function, many functional genomic studies have profiled transcriptomic data of individuals with multiple samples of different origins [1, 2], diverse cell types [3, 4], and various time points [5–7]. These measurements of gene expression levels from multiple samples of the same individual are correlated in nature. To analyze the correlated gene expression levels under complex structures, linear mixed-effects models for genes are needed. Due to the constrain of availability, financial resources, or even practical considerations, small number of individuals are commonly used for many studies, which can lead to undesired low power for detecting differential expression between conditions when genes are analyzed separately using linear mixed-effects models. Therefore, variance shrinkage over the range of genes’ expression levels is necessary in order to improve power. However, currently there is a lack of methods that use variance shrinkage techniques for linear mixed-effects models.

Several variance shrinkage methods through moderation under hierarchical Bayes framework have been proposed for detecting differential expression with microarray or RNA-Seq data [8–11]. These methods are based on variance shrinkage of residual errors of linear models with only fixed effects, but the power gain relative to other test methods without variance shrinkage is significant. However, use of these models in designs with correlation structures will lead to biased estimation of gene variances, thus will result in inflated false positive and false negative rates.

There are two variance shrinkage methods that try to address the correlation issue. Limma method [12] incorporates correlation estimates of replicates in linear modeling, but it enforces a common correlation for all genes, which works well for within-array replicates as originally planned, but is risky for between-array replicates. Dream method [13] tries to solve this issue in the setting of linear mixed-effects models. This method shrinks residual variances only, while variance estimates of random effects are scaled to residual variances before and after shrinkage. The limitation of this approach is that variance shrinkage of random effects is ignored by using the same shrinkage for both random and residual variances over the range of genes’ expression levels.

Toward this end, we implemented a novel procedure that allows us to shrink both types of variation independently through moderated t-methods under linear mixed-effects models. Our method assumes that variances of both residual errors and random effects have different mean functional forms over the range of genes’ expression levels. Then testing for differential expression between conditions is based on the combined variance shrinkage estimators. Simulations were performed to evaluate the proposed procedure. A real gene expression experiment on alopecia areata treatment on mouse skin samples was used as a case study.

## Methods

### Linear mixed-effects model

For each gene, *Y* denotes expression levels of all samples, which are normally distributed. The linear mixed-effects model (LMM) is formulated as

$$Y = X\beta + Z\gamma + \epsilon,$$

where *β* are fixed-effects, *γ* are random-effects, and *ε* are residual errors with *Var*(*γ*)=*G* and *Var*(*ε*)=*R*. *γ* and *ε* are independent. *Y* has mean *Xβ* and variance *ZGZ*^′^+*R*.

The hypothesis for testing fixed-effects is

$$H_{0}: L\beta=0 \text{ vs.} H_{1}: L\beta \neq 0$$

Then the t-statistic for the hypothesis test is

$$T=\frac{L\hat{\beta}}{\sqrt{L\hat{C}L^{\prime}}} $$

where $\hat {\beta }=(X^{\prime }\hat {V}^{-1}X)^{-}X^{\prime }\hat {V}^{-1}Y$, $C=(X^{\prime }\hat {V}^{-1}X)^{-}$.

### Hierarchical model

Following our previously published fully moderated t-statistic (FMT) method [10], a hierarchical Bayes model is assumed for residual variances or variance estimates of a random effect from a linear mixed-effects model of log transformed expression data.

Residual variance for a gene *g*, $s_{e_{g}}^{2}$, is assumed to follow a scaled Chi-square distribution with $d_{e_{g}}$ degrees of freedom:

$$\begin{array}{@{}rcl@{}} s_{e_{g}}^{2} | \sigma_{e_{g}}^{2} & \sim & \frac{\sigma_{e_{g}}^{2}}{d_{e_{g}}}\chi_{d_{e_{g}}}^{2}, \end{array} $$

where $\sigma _{e_{g}}^{2}$ is the variance of residual error, which is assigned a scaled inverse Chi-square prior:

1 $$\begin{array}{@{}rcl@{}} \frac{1}{\sigma_{e_{g}}^{2}} & \sim & \frac{1}{d_{0e_{g}}s_{0e_{g}}^{2}}\chi_{d_{0e_{g}}}^{2}.  \end{array} $$

$d_{0e_{g}}$ and $s_{0e_{g}}^{2}$ are the prior degrees of freedom and location, respectively. Under this model, the posterior mean of $\sigma _{e_{g}}^{2}$, given $s_{e_{g}}^{2}$, is

$$\begin{array}{@{}rcl@{}} \stackrel{\sim}{s}_{e_{g}}^{2} & = & \frac{d_{0e_{g}}s_{0e_{g}}^{2}+d_{e_{g}}s_{e_{g}}^{2}}{d_{0e_{g}}+d_{e_{g}}}. \end{array} $$

Similarly, variance estimate of a random effect for a gene *g*, $s_{r_{g}}^{2}$, is assumed to follow a scaled Chi-square distribution with $d_{r_{g}}$ degrees of freedom:

$$\begin{array}{@{}rcl@{}} s_{r_{g}}^{2} | \sigma_{r_{g}}^{2} & \sim & \frac{\sigma_{r_{g}}^{2}}{d_{r_{g}}}\chi_{d_{r_{g}}}^{2}, \end{array} $$

where $\sigma _{r_{g}}^{2}$ is the variance of a random effect, which is also assigned a scaled inverse Chi-square prior:

2 $$\begin{array}{@{}rcl@{}} \frac{1}{\sigma_{r_{g}}^{2}} & \sim & \frac{1}{d_{0r_{g}}s_{0r_{g}}^{2}}\chi_{d_{0r_{g}}}^{2}.  \end{array} $$

$d_{0r_{g}}$ and $s_{0r_{g}}^{2}$ are the prior degrees of freedom and location, respectively. Under this model, the posterior mean of $\sigma _{r_{g}}^{2}$, given $s_{r_{g}}^{2}$, is

$$\begin{array}{@{}rcl@{}} \stackrel{\sim}{s}_{r_{g}}^{2} & = & \frac{d_{0r_{g}}s_{0r_{g}}^{2}+d_{r_{g}}s_{r_{g}}^{2}}{d_{0r_{g}}+d_{r_{g}}}. \end{array} $$

Then the denominator of the moderated t-statistic is

$$\begin{array}{@{}rcl@{}} \stackrel{\sim}{t}_{g} & = & \frac{L\hat{\beta}_{g}}{\sqrt{L\stackrel{\sim}{C}_{g}L^{\prime}}}.  \end{array} $$

Variance components method [14] or Welch-Satterthwaite method [15] is used to calculate an approximation to the degrees of freedom for the linear combinations of independent posterior variance estimates $\stackrel {\sim }{s}_{e_{g}}^{2}$ and $\stackrel {\sim }{s}_{r_{g}}^{2}$.

### Estimation of hyper-parameters

Hyper-parameter estimation procedures for $\sigma _{e_{g}}^{2}$ and $\sigma _{r_{g}}^{2}$ are performed separately. The hyperparameters $d_{0e_{g}}$ and $s_{0e_{g}}^{2}$ as well as $d_{0r_{g}}$ and $s_{0r_{g}}^{2}$ are estimated by an empirical Bayes approach implemented in our FMT method [10]. The accuracy of hyper-parameter estimation (in terms of bias and variance) can be approximated by using the empirical distribution of hyper-parameters’ estimates generated from multiple simulation runs, which is demonstrated in our FMT method paper.

### False positive control

With thousands of genes available in genome-wide profiling studies, multiplicity adjustment is needed for false positive error control. While the Bonferroni method controls the family-wise error rate (probability of one or more false rejections among all comparisons), it is well-known to be a conservative method for genome-wide profiling studies. A less conservative procedure (the extended interpretation of the Bonferroni method) that controls the expected mean number of false positives was used for multiplicity adjustment throughout this paper [16]. This procedure controls the per family error rate (PFER). It is as powerful as the Benjamimi-Hochberg (BH) FDR control procedure, but has better stability compared to the BH FDR control procedure. In simulations, the nominal level for the false positive error control is set as the PFER.

### Simulation procedures

- *1*: For 12,000 genes (*g*=1,2,⋯,12,000), simulate average log expression, *α*_*g*_, from a three parameter log-normal distribution: *ln*(*α*_*g*_−3.75)∼*N*(1.35,0.35).
- **2**: For $\sigma _{e_{g}}^{2}$ of gene *g*, calculate the gene-specific prior location $s_{0e_{g}}^{2}$ and prior degrees of freedom $d_{0e_{g}}$ as a function of the average log expression values (*α*_*g*_) obtained from Step 1. The prior location $s_{0e_{g}}^{2}$ and prior degrees of freedom $d_{0e_{g}}$ are modeled as:
  $${{}{\begin{aligned}s_{0e_{g}}^{2} & = 0.2 e^{-1.2(\alpha_{g}-4.6)}+0.05 \\ d_{0e_{g}} & = 1.5 \sqrt{-1.1e^{0.1(\alpha_{g}-6)} + 0.6(\alpha_{g}-7)^{2} + 20} + e^{0.25(\alpha_{g}-14)}\! + 2 \end{aligned}}} $$
  For $\sigma _{r_{g}}^{2}$ of gene *g*, calculate the gene-specific prior location $s_{0r_{g}}^{2}$ and prior degrees of freedom $d_{0r_{g}}$ as a function of the average log expression values (*α*_*g*_) obtained from Step 1. The prior location $s_{0r_{g}}^{2}$ and prior degrees of freedom $d_{0r_{g}}$ are modeled as:
  $${{}{\begin{aligned} s_{0r_{g}}^{2} & = 0.5 e^{-0.8(\alpha_{g}-5.25)}+0.1 \\ d_{0r_{g}} & = 1.2 \sqrt{-1.1e^{0.1(\alpha_{g}-6)} + 0.6(\alpha_{g}-7)^{2} + 20} + e^{0.25(\alpha_{g}-14)}\! + 2 \end{aligned}}} $$
- **3**: For gene *g*, simulate $\sigma _{e_{g}}^{2}$ and $\sigma _{r_{g}}^{2}$ from an inverse Chi-square distribution (Eqs. 1 and 2) using the hyper-parameters from Step 2.
- **4**: For gene *g*, simulate random effect *S*_*i*_ from $N(0,\sigma _{r_{g}}^{2})$.
- **5**: Among the 12,000 expressed genes, 500 genes are simulated to be differentially expressed using the following steps:
  - Simulate mean log-differences *μ*_*g*_ from *N*(0,2) for 500 differentially expressed genes.
  - Simulate expression data for each replicate in group 1 from $N(\alpha _{g}+\mu _{g}/2+S_{i},\sigma _{e_{g}}^{2})$ and for each replicate in group 2 from $N(\alpha _{g}-\mu _{g}/2+S_{i},\sigma _{e_{g}}^{2})$.
- **6**: For the remaining 11,500 expressed genes, simulate expression data for each replicate from $N(\alpha _{g}+S_{i},\sigma _{e_{g}}^{2})$.

## Results

### Simulation

#### Parameter setting

For simulation, we chose a simple design for gene expression experiment without loss of generality. We assumed two cohort groups with each cohort having *n*_*s*_ biological replicates (subjects) and each subject having *n*_*r*_ technical replicates. Our main interest is to compare gene expression differences between two cohort groups for each gene. We simulated gene expression data under linear mixed-effects models, where subject is a random effect and variation due to technical replicates was included in the residual errors.

Expression levels of 12,000 genes were simulated independently, among which 500 genes were simulated with differential expression between two cohort groups (see above Simulation Procedures). Simulation models were based on model assumptions of our published FMT method, which allows hyper-parameters to vary as a function of genes’ expression levels. All parameters were set to resemble real gene expression arrays. Under this setting, we generated data sets for 13 different biological replicates (3-20 in each cohort group) and 6 different technical replicates (2-11) within each biological replicate. One hundred simulation runs were conducted for each simulation scenario.

#### Methods comparison

We compared the proposed method (FMT in LMM) to Limma [12] and Dream methods [13]. The Limma method calculates a common correlation coefficient between technical replicates for all genes to account for the correlation of repeated measurements within subjects in a linear fixed-effects model setting. The Dream method uses a linear mixed-effects model, where variance estimates of random subject effect is standardized to residual variances, then residual variances were modeled as a function of average of expression levels. In our proposed method, we used two ways to estimate the degrees of freedom of the moderated t-statistic in Eq. 3. The first approach is the variance components method (VC), which divides the residual degrees of freedom into between-subject and within-subject portions based on variance decomposition and assigns the between-subject degrees of freedom to the fixed effects if subjects are nested in the fixed effects. The second approach is the Welch-Satterthwaite method (Sat), which uses an approximation of the effective degrees of freedom of a linear combination of independent variance estimates. We also compared our method to the ordinary t-test method (OT) in LMM, which does not perform variance shrinkage to the variance estimates.

#### Power and false positives

**Fig. 1:**
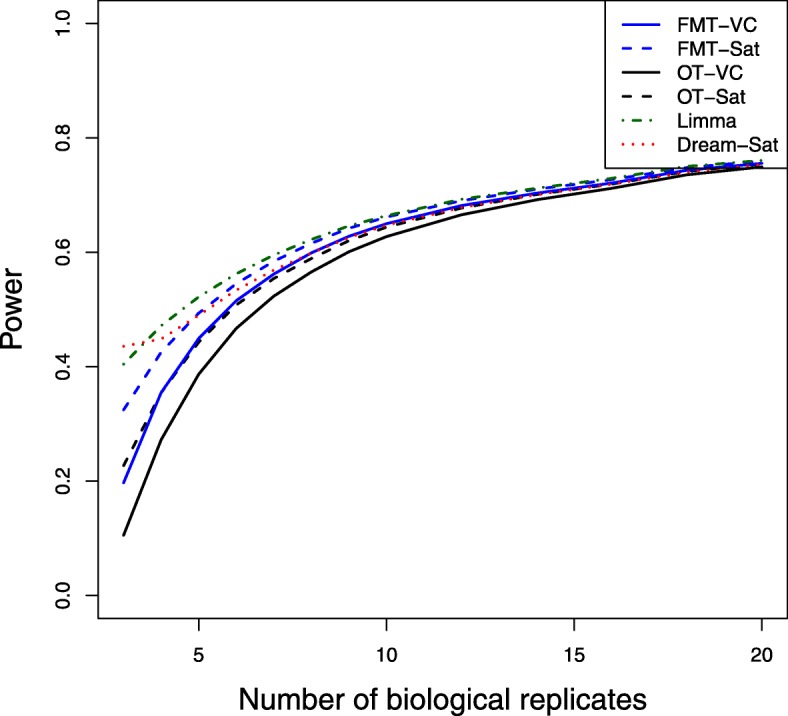
Power by numbers of biological replicates. Power was averaged over 100 simulation runs for 13 different sample sizes of biological replicates where the number of technical replicates equals 2 and the expected mean number of false positives equals 5. Different methods were used for the degrees of freedom approximation: FMT-VC (solid blue) is the fully moderated t-test with the variance components method; FMT-Sat (dotted blue) is the fully moderated t-test with the Welch-Satterthwaite method; OT-VC (solid black) is the ordinary t-test with the variance components method; OT-Sat (dotted black) is the ordinary t-test with the Welch-Satterthwaite method; Limma (dotted dark green) is the Limma method with replicates’ correlation estimation; Dream-Sat (dotted red) is the Dream method with the Welch-Satterthwaite method

**Fig. 2:**
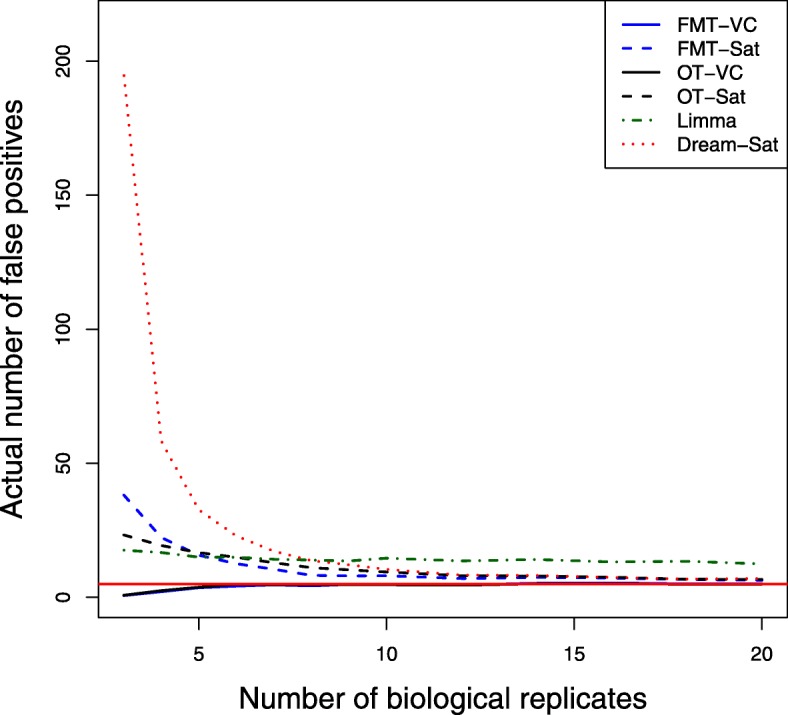
False positives by numbers of biological replicates. Actural number of false positives was averaged over 100 simulation runs for 13 different sample sizes of biological replicates where the number of technical replicates equals 2 and the expected mean number of false positives equals 5. Different methods were used for the degrees of freedom approximation: FMT-VC (solid blue) is the fully moderated t-test with the variance components method; FMT-Sat (dotted blue) is the fully moderated t-test with the Welch-Satterthwaite method; OT-VC (solid black) is the ordinary t-test with the variance components method; OT-Sat (dotted black) is the ordinary t-test with the Welch-Satterthwaite method; Limma (dotted dark green) is the Limma method with replicates’ correlation estimation; Dream-Sat (dotted red) is the Dream method with the Welch-Satterthwaite method

Power averaged over 100 simulation runs are shown in Fig. 1, where power is plotted against numbers of biological replicates (3-20) for 2 technical replicates and 5 expected mean number of false positives. The Limma method has the best power except at the small number of biological replicates and OT-VC has the lowest power. The power difference between all methods goes down when the number of biological replicates increases. For both FMT and OT methods, the Welch-Satterthwaite method results in better power than the variance components method. Fig. 2 shows the actual number of false positives over numbers of biological replicates for 2 technical replicates and 5 expected mean number of false positives. The Dream method has the largest actual number of false positives, and it spikes more than 100 fold higher than the controlled level when the number of biological replicates is relatively small and drops dramatically when the number of biological replicates increases. The Limma method has inflated actual number of false positives as well, but it is relatively stable over numbers of biological replicates. Only FMT-VC and OT-VC maintain the nominal level of false positive error control at each biological replicate level. Similar patterns are observed for higher numbers of technical replicates.

**Fig. 3:**
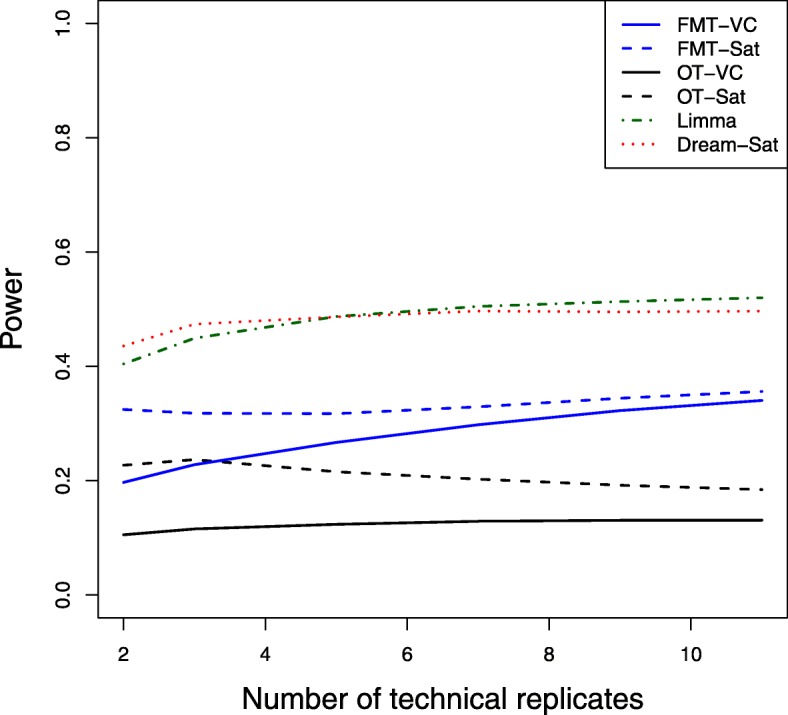
Power by numbers of technical replicates. Power was averaged over 100 simulation runs for 6 different sample sizes of technical replicates where the number of biological replicates equals 3 and the expected mean number of false positives equals 5. Different methods were used for the degrees of freedom approximation: FMT-VC (solid blue) is the fully moderated t-test with the variance components method; FMT-Sat (dotted blue) is the fully moderated t-test with the Welch-Satterthwaite method; OT-VC (solid black) is the ordinary t-test with the variance components method; OT-Sat (dotted black) is the ordinary t-test with the Welch-Satterthwaite method; Limma (dotted dark green) is the Limma method with replicates’ correlation estimation; Dream-Sat (dotted red) is the Dream method with the Welch-Satterthwaite method

**Fig. 4:**
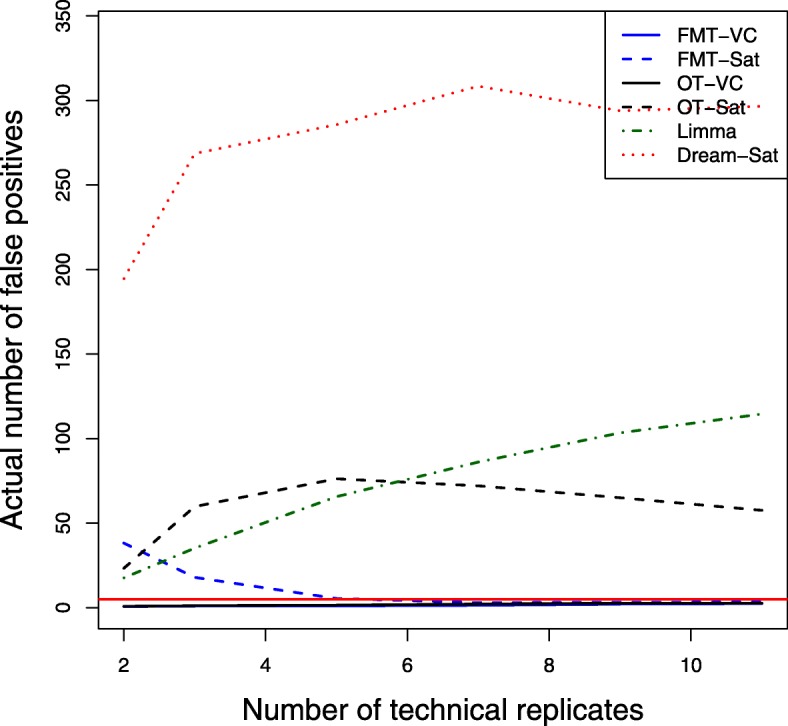
False positives by numbers of technical replicates. Actual number of false positives was averaged over 100 simulation runs for 6 different sample sizes of technical replicates where the number of biological replicates equals 3 and the expected mean number of false positives equals 5. Different methods were used for the degrees of freedom approximation: FMT-VC (solid blue) is the fully moderated t-test with the variance components method; FMT-Sat (dotted blue) is the fully moderated t-test with the Welch-Satterthwaite method; OT-VC (solid black) is the ordinary t-test with the variance components method; OT-Sat (dotted black) is the ordinary t-test with the Welch-Satterthwaite method; Limma (dotted dark green) is the Limma method with replicates’ correlation estimation; Dream-Sat (dotted red) is the Dream method with the Welch-Satterthwaite method

Power averaged over 100 simulation runs is plotted against numbers of technical replicates (2-11) for 3 biological replicate and 5 expected number of false positives as shown in Fig. 3. The Limma and Dream methods have higher power than other methods. The power differences between methods seem stable across the range of technical replicates. Figure 4 shows the actual number of false positives by technical replicates for 3 biological replicates and 5 expected number of false positives. Similarly, FMT-VC and OT-VC maintain the desired false positive error control at each technical replicate number. When the number of technical replicates is bigger than 4, the FMT-Sat method maintains the desired false positive error control, while the other three methods still fail to maintain false positives at the nominal level. For both Limma and Dream methods, the actual number of false positives increases with larger numbers of technical replicates. Similar patterns are observed for higher numbers of biological replicates. We believe that the ultrally inflated actual number of false positives for both Limma and Dream methods contributes to the higher power compared to both FMT and OT methods.

#### Gene correlation

To understand effects of gene correlation on the tests’ performance, we also performed simulations using a block-diagonal gene correlation structure. We randomly assigned genes into blocks of size 10, where gene correlations inside blocks are fixed at 0.5 while gene correlations between blocks are set to 0. Results on power and actual number of false positives under the gene correlation structure are highly similar to those under gene independence. Supplemental plots are provided in Additional file 1.

### A case study

**Fig. 5:**
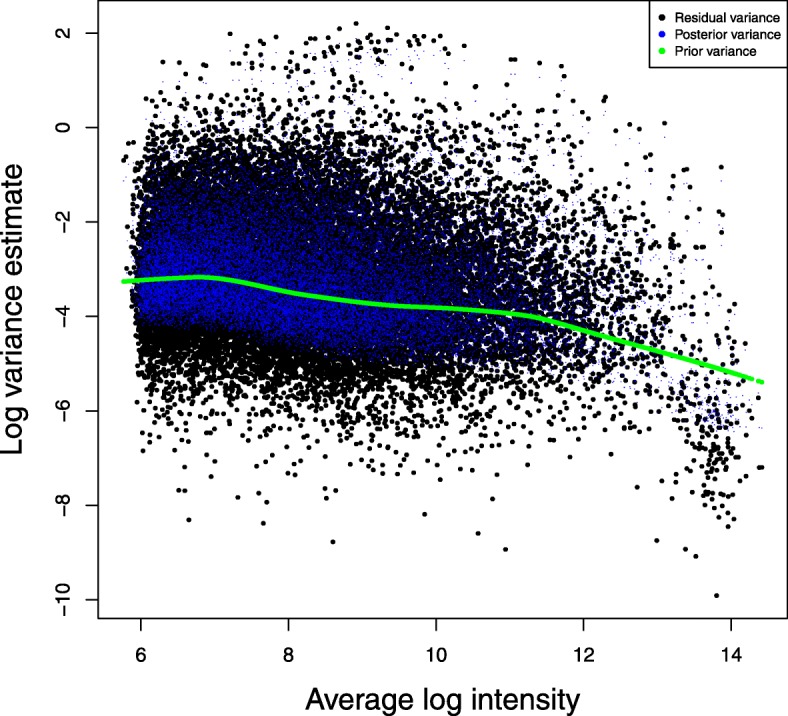
Residual variance in the case study. FMT-VC method was applied to analyze the microarray data in the case study. Black dots represent per-gene residual variance. Blue dots represent per-gene posterior of residual variance. Green dots represent per-gene prior of residual variance

**Fig. 6:**
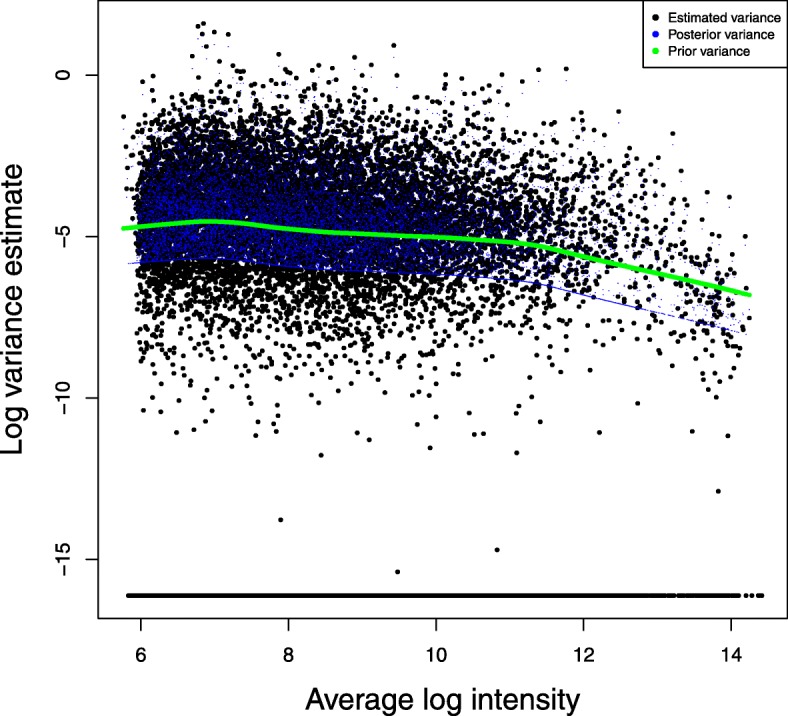
Variance estimates of random effect in the case study. FMT-VC method was applied to analyze the microarray data in the case study. Black dots represent per-gene variance estimates of random effect. Blue dots represent per-gene posterior variance estimates of random effect. Green dots represent per-gene prior variance estimates of random effect

**Fig. 7:**
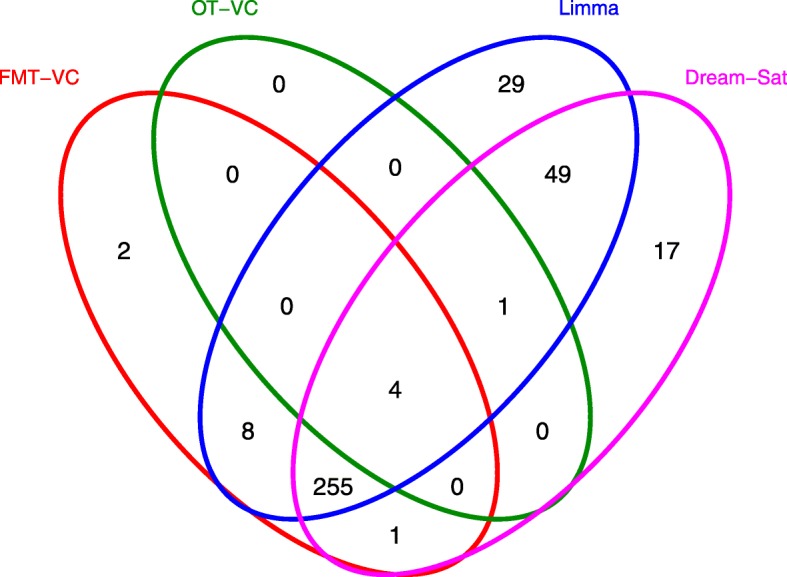
Venn diagram of significant genes of week 12 in the case study. Four methods were applied to analyze the microarray data in the case study. Lists of significant gene of week 12 were generated and compared among methods for 5 expected number of false positives. Red circle is for FMT-VC method. Green circle is for OT-VC method. Blue circle is for Limma method. Pink circle is for Dream-Sat method

To demonstrate the performance of the proposed method for detecting differential expression under linear mixed-effects models, we applied it to a real microarray study published in Nature Medicine in 2014 by Xing et al. [17]. Microarray data were deposited in the GEO database with an access number GSE45514. This study used Affymetrix Mouse Genome 430 2.0 Array on mouse skin samples taken at different time points (weeks 6 and 12) from C3H/HeJ mice treated with topical JAK inhibitors ruxolitinib (Jak1), tofacitinib (Jak3) or control (PBS). Three skin samples were taken at each time point from 3 mice for each treatment. We focused on the comparison between two treatments (J3 vs. PBS), and we analyzed the data using our FMT-VC linear mixed-effects model where mouse was treated as a random effect. We compared the FMT-VC method with other methods as done in simulation studies. Figures 5 and 6 show the fitted values of residual variances and variance estimates of random mouse effect. As shown in the venn-diagram in Fig. 7, lists of significant genes of week 12 at the PFER cutoff value of 5 expected mean number of false positives are compared among methods. There are several genes detected only by the Limma and Dream methods, but these genes are most likely to be false positives since our simulation studies demonstrate the ultrally inflated false positive rate for both methods especially when the number of biological replicates is small. The few significant genes for OT-VC method are mainly due to low power of that method.

## Discussion

Differential expression analysis through moderated t-tests has become common practice in genomic studies for the past decade. But most moderated t-tests are limited to simple designs with the usage of linear fixed-effects models. Recent efforts have been made to accommodate designs associated with complex correlation structures using LMM. In this paper, we proposed a novel approach to address the need of applying moderated methods in LMM and compared our method with two other current methods. Our proposed method is very flexible because we allow different shrinkage over the range of expression levels for each variance component. However the Limma and Dream methods can only do shrinkage of residual variances over the range of expression levels.

We have employed two approaches for approximating degrees of freedom of moderated t-statistic (the variance components method and the Welch-Satterthwaite method) for both FMT and OT methods in LMM for testing differential expression. The Welch-Satterthwaite method uses all variance estimates to approximate degrees of freedom, but the VC method only accounts for between-subject variation and ignores within-subject variation from the total degrees of freedom when fixed effects change across subjects. The Welch-Satterthwaite method inflates type I error and fails to maintain false positive error control at the nominal level, but the VC method performs proper control for false positive errors. In addition, FMT-VC is more powerful than OT-VC mainly due to moderation.

The performance of the proposed method was demonstrated through a case study with a simple design. However it can be applied to comprehensive designs including multi-factors or multi-levels in linear mixed-effects models. The proposed method can also be applied to data sets that measure gene expression by other technologies, *e.g.* RNA-Seq, protein expression, or quantities of small molecules.

## Conclusions

Current moderated-t methods are limited to shrinking only the residual variance of linear models. In this paper, we proposed an approach to allow variance shrinkage of both residual error and random effects using moderated t-statistic under LMM. The gain in power through moderation and proper control of false positive errors was demonstrated by simulation studies. We also applied the proposed method to a real gene expression data set.

## Supplementary information

**Additional file 1** This pdf file contains all supplementary figures referenced in results section.

## Data Availability

The microarray data set used in the case study is from publicly available repositories. R package fmt used in the analysis is available from CRAN. R code for the case study is available from the corresponding author.

## Abbreviations

BH: Benjamimi-Hochberg
FDR: False discovery rate
FMT: Fully moderated t-statistic
LMM: Linar mixed-effects models
PFER: Per family error rate
