# Supplementary figures and images for: Fully moderated t-statistic in linear modeling of mixed effects for differential expression analysis
Source: BMC Bioinformatics. 2019 Dec 20;20(Suppl 24):675. doi: 10.1186/s12859-019-3248-9 (PMC6923909; doi:10.1186/s12859-019-3248-9)

## Supplemental Figures:

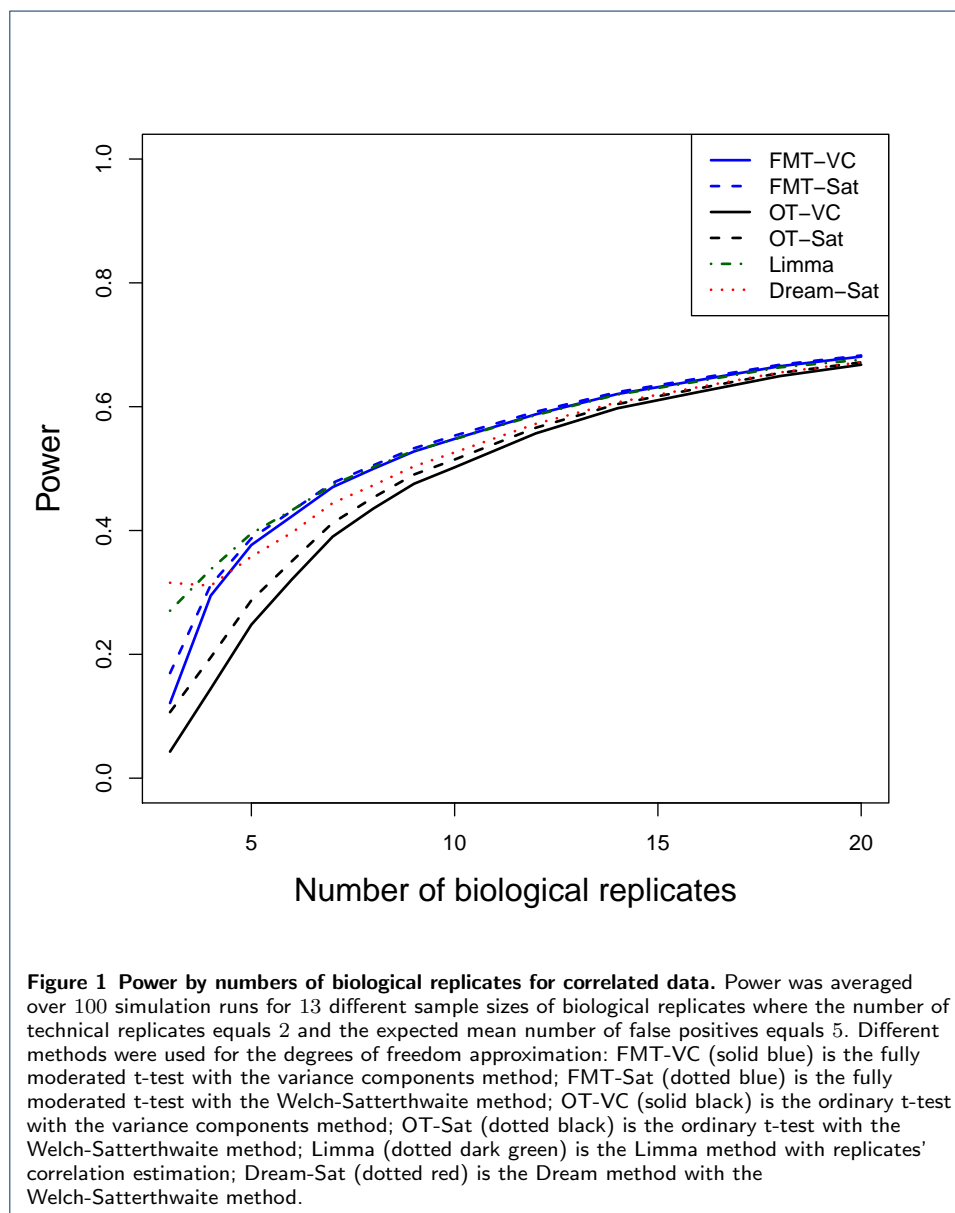

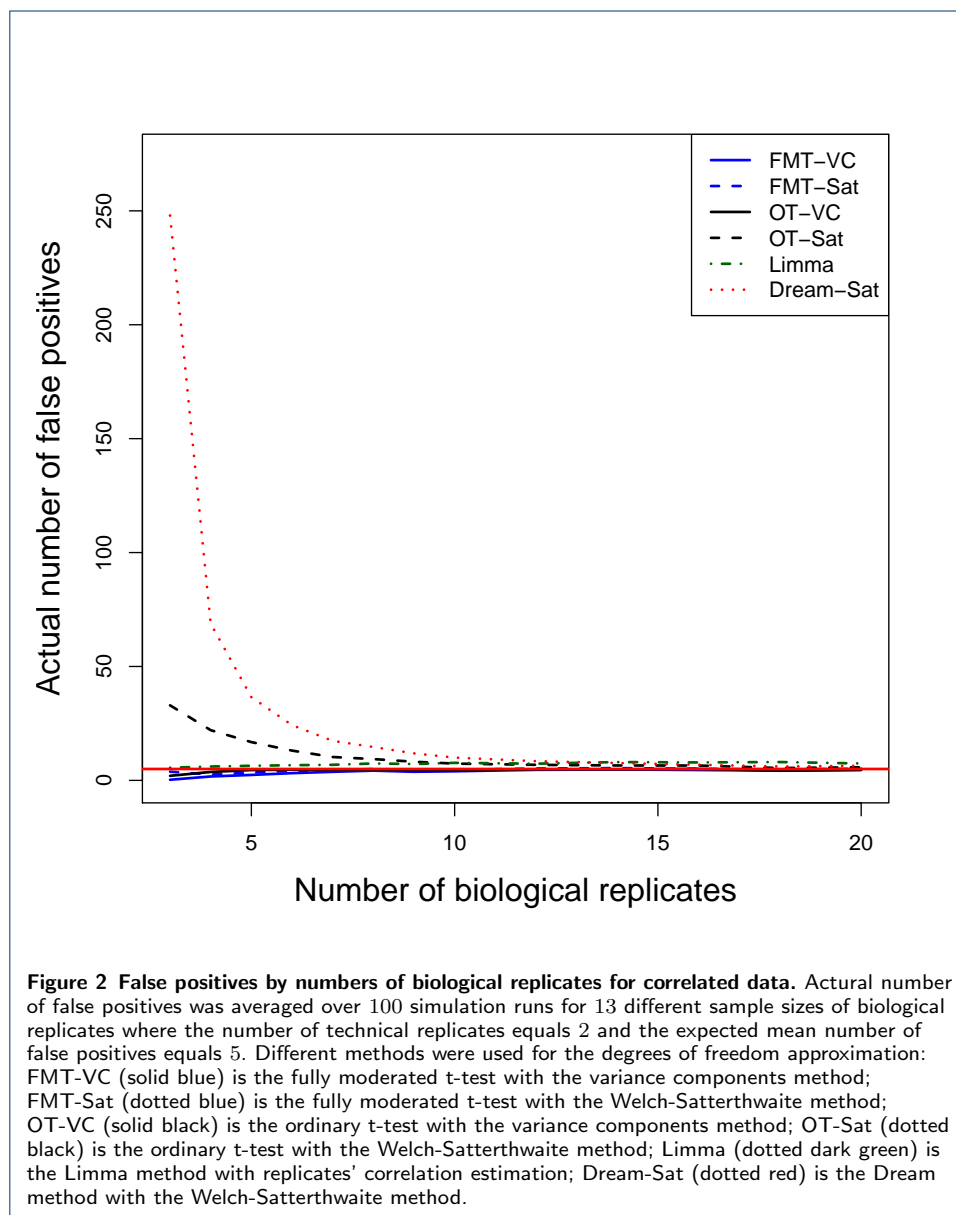

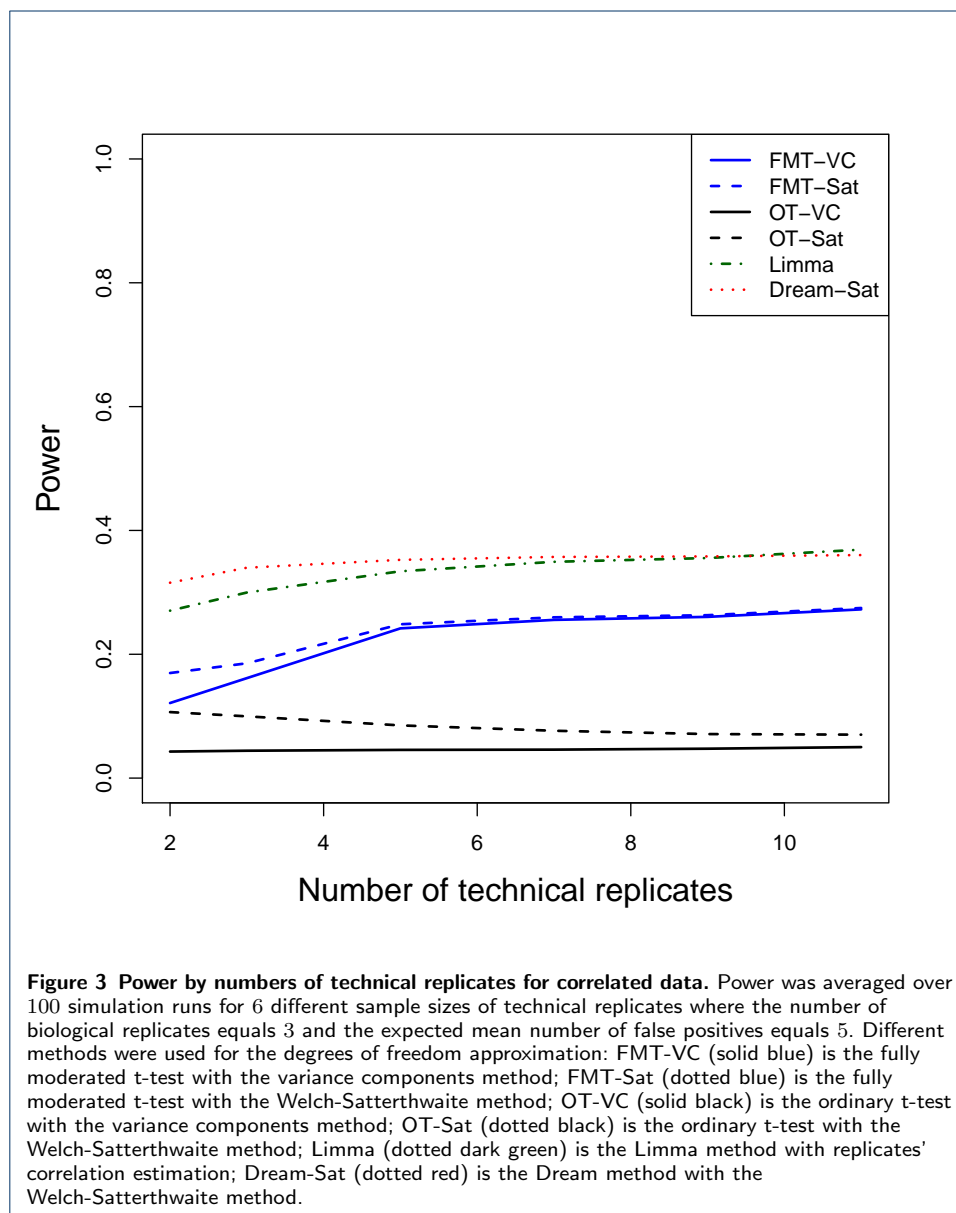

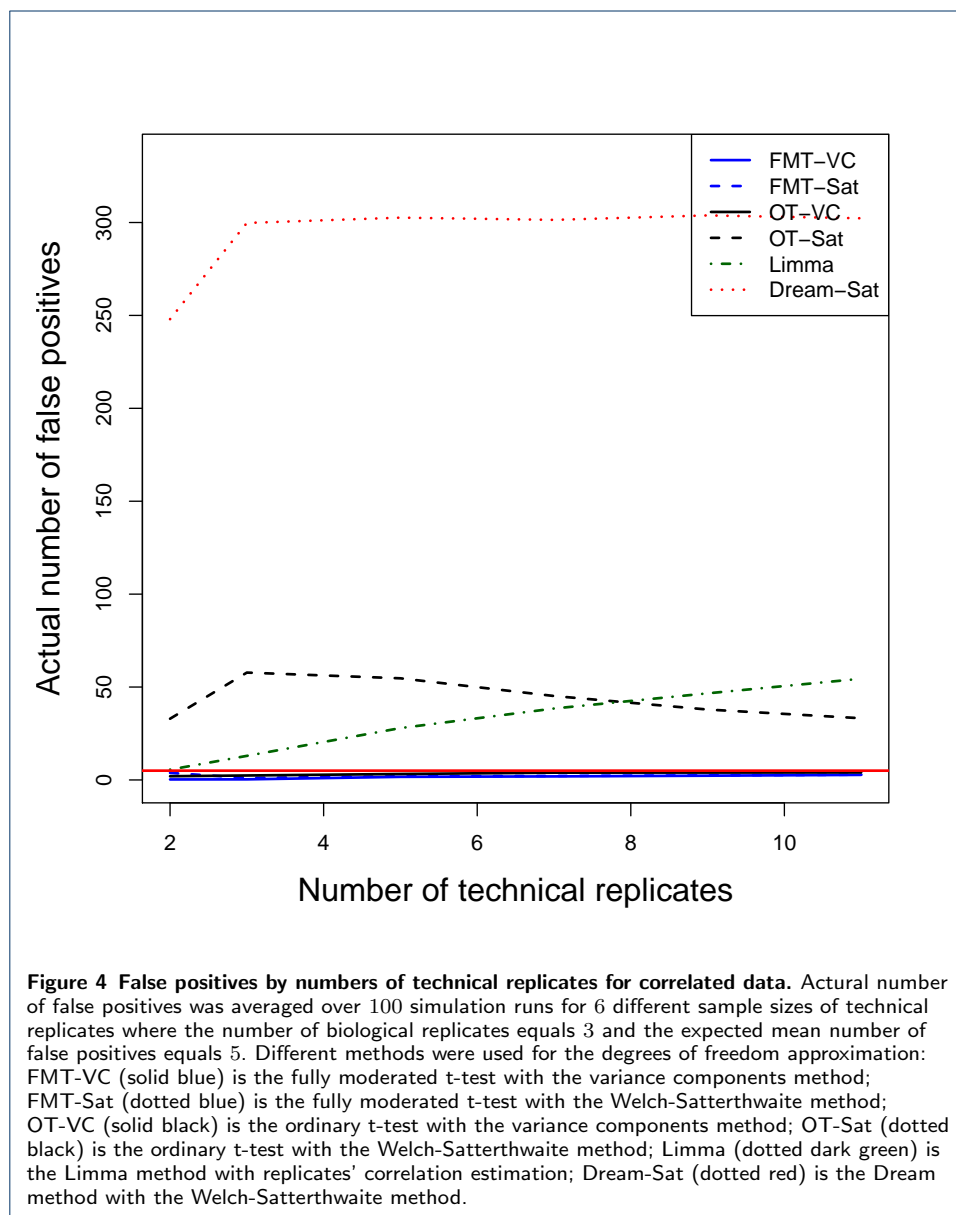

Supplement: Supplementary file 1 — Additional file 1 This pdf file contains all supplementary figures referenced in results section. [file 12859_2019_3248_MOESM1_ESM.pdf]
